# Two Major Autoantibody Clusters in Systemic Lupus Erythematosus

**DOI:** 10.1371/journal.pone.0032001

**Published:** 2012-02-21

**Authors:** Kathryn H. Ching, Peter D. Burbelo, Christopher Tipton, Chungwen Wei, Michelle Petri, Ignacio Sanz, Michael J. Iadarola

**Affiliations:** 1 Neurobiology and Pain Therapeutics Section, Laboratory of Sensory Biology, National Institute of Dental and Craniofacial Research, Bethesda, Maryland, United States of America; 2 Division of Allergy, Immunology and Rheumatology, Department of Medicine, University of Rochester Medical Center, Rochester, New York, United States of America; 3 Division of Rheumatology, Department of Medicine, Johns Hopkins University School of Medicine, Baltimore, Maryland, United States of America; Carl-Gustav Carus Technical University-Dresden, Germany

## Abstract

Systemic lupus erythematosus is a chronic autoimmune disease of complex clinical presentation and etiology and is likely influenced by numerous genetic and environmental factors. While a large number of susceptibility genes have been identified, the production of antibodies against a distinct subset of nuclear proteins remains a primary distinguishing characteristic in disease diagnosis. However, the utility of autoantibody biomarkers for disease sub-classification and grouping remains elusive, in part, because of the difficulty in large scale profiling using a uniform, quantitative platform. In the present study serological profiles of several known SLE antigens, including Sm-D3, RNP-A, RNP-70k, Ro52, Ro60, and La, as well as other cytokine and neuronal antigens were obtained using the luciferase immunoprecipitation systems (LIPS) approach. The resulting autoantibody profiles revealed that 88% of a pilot cohort and 98% of a second independent cohort segregated into one of two distinct clusters defined by autoantibodies against Sm/anti-RNP or Ro/La autoantigens, proteins often involved in RNA binding activities. The Sm/RNP cluster was associated with a higher prevalence of serositis in comparison to the Ro/La cluster (*P* = 0.0022). However, from the available clinical information, no other clinical characteristics were associated with either cluster. In contrast, evaluation of autoantibodies on an individual basis revealed an association between anti-Sm (*P* = 0.006), RNP-A (*P* = 0.018) and RNP-70k (*P* = 0.010) autoantibodies and mucocutaneous symptoms and between anti-RNP-70k and musculoskeletal manifestations (*P* = 0.059). Serologically active, but clinically quiescent disease also had a higher prevalence of anti-IFN-α autoantibodies. Based on our findings that most SLE patients belong to either a Sm/RNP or Ro/La autoantigen cluster, these results suggest the possibility that alterations in RNA-RNA-binding protein interactions may play a critical role in triggering and/or the pathogenesis of SLE.

## Introduction

Systemic lupus erythematosis (SLE) is an autoimmune inflammatory disease characterized by interferon and complement activation, autoantibodies, and tissue destruction involving multiple organ systems [1]. In addition, activation of type I interferons is also prevalent in SLE and may be associated with distinct autoantibody profiles [2]. Common clinical symptoms in SLE include rash, nephritis, central nervous system disease, thrombocytopenia and musculoskeletal manifestations. SLE often occurs in women between 20–40 years of age and has strong genetic and environmental components [3], [4], [5]. Despite genetic studies identifying a large number of susceptibility genes including key immunological regulators, such as BANK1, TNFAIP3, ITGAM, PD1 and STAT4 [5], [6], [7], exploiting this information for identifying and stratifying clinical subsets of SLE has been largely unsuccessful. The use of genetic markers to identify and stratify clinical subsets is hampered by genetic complexity and the high frequency of many of the SLE susceptibility alleles in the general population.

In contrast to genetic analyses, autoantibodies represent a major diagnostic feature of SLE and can provide clues to pathological processes in various tissues. Although a very large number of autoantibodies have been described in SLE [8], only anti-double stranded DNA (dsDNA), Smith (Sm) and phospholipid (PL) autoantibodies are part of the classification criteria outlined by the American College of Rheumatology, although it should be noted that anti-PL (aPL) autoantibodies are not specific for SLE [9]. Similarly, other major nuclear and cytoplasmic target antigens, including several ribonuclear proteins (RNP), the RNA binding proteins Ro52 and Ro60 and the 48 kDa protein La, while prevalent in SLE, are not specific for the disease. In recent years, a significant effort has been directed at understanding the relationship between autoantibody profiles and specific disease subsets. For example, in one study, anti-Ro and anti-La autoantibodies, which are prevalent in Sjogren's Syndrome, were associated with an increased risk of sicca symptoms of dry mouth and eyes [10], [11]. Anti-La autoantibodies have also been associated with less severe disease and a reduced risk of lupus nephritis [12]. Autoantibodies against Sm and RNP are often found together and were associated with a higher incidence of Raynaud's syndrome and leukopenia [10]. Lupus anticoagulant (LAC) and anti-cardiolipin (cL) antibodies have been observed to correlate with an increased risk of venous thrombosis [13]. The numerous and often inconsistent clinical associations reported for autoantibodies observed in lupus may reflect differences in method of detection and composition of the patient cohort. These and other findings suggest that further improvements in SLE antibody profiles including quantitative assessment of autoantibody titers, increasing the spectrum of targets examined, and assay simplification might lead to improved diagnosis, classification, therapeutic intervention, and prognosis.

To test whether high resolution profiling of lupus autoantibodies would provide data for clinically informative clustering, a solution phase assay format called luciferase immunoprecipitation assay systems (LIPS) was used. LIPS employs the light emitting *Renilla* luciferase (Ruc) enzyme genetically fused to potential protein or peptide antigens. This provides a uniform platform for detection of autoantibodies against various tagged proteins. LIPS is quantitative, linear up to 7 log units, and in previous studies in several different autoimmune conditions yielded higher sensitivity and specificity and/or a larger dynamic range than existing ELISA or radiobinding assays [14].

In this study, a pilot and second cohort of SLE patients and control serum samples were evaluated against a panel of autoantigens including seven nuclear antigens, five cytokines, and five CNS-enriched proteins. We also evaluated a potentially new test for lupus autoantibodies by combining six of the major autoimmune targets into one assay. Analysis of the autoantibody profiles, in conjunction with available clinical information, revealed several associations between autoantibodies and specific clinical manifestations. We also observed a high frequency of anti-IFN-ω autoantibodies in the SLE cohort, which correlated with high titer anti-Sm, anti-RNP-A and anti-RNP-70k autoantibodies. Additionally, we identified two distinct patient clusters based on titer ratios that dichotomize the population with at least one clinical symptom, serositis, clearly associating with the validation cohort. The data presented suggest multifactorial roles for autoantigens in lupus, and emphasize the need for further refinements in autoantibody testing and more intensive profiling in order to more thoroughly understand and treat this disease.

## Materials and Methods

### Ethics Statement

Serum samples from SLE patients and healthy volunteers were obtained from the Department of Rheumatology, University of Rochester Medical Center and the Division of Rheumatology, The Johns Hopkins University School of Medicine. All studies were conducted, and all samples were obtained with written, informed consent under Institutional Review Board approved protocols from the University of Rochester Medical Center and The Johns Hopkins Medical Center.

### Patients and serum samples

All SLE patients fulfilled at least four of the American College of Rheumatology criteria for diagnosis. The initial training set consisted of 18 healthy volunteers and 76 SLE patients. The independent validation cohort consisted of 15 new healthy controls and 129 SLE patients. Sera were stored at −80°C, then diluted 1∶10 in buffer A (50 mM Tris (pH 7.4), 100 mM NaCl, 5 mM MgCl_2_, 1% Triton X-100 and a protease inhibitor cocktail (Roche)) and stored at −20°C prior to use.

### Generation and expression of Ruc-antigen fusion proteins

Several *Renilla* luciferase (Ruc) C-terminal fusion proteins representing known SLE targets including Ro52, Ro60 and La have been previously described [15], [16]. The GenBank accession numbers and exact amino acids (aa) used for these target antigens are as follows: La (NP_003133.1; aa 2–408), Ro52 (NP_003132.2 ; aa 2–276), Ro60 (NP_004591.2|; aa 244–538), Sm-D3 (NP_004166.1|; aa 2–126), snRNP A1 (NP_004587.1|; aa 1–282, referred to as RNP-A in the manuscript), snRNP 70k (NP_003080| ; aa 1–437, referred to as RNP-70k in the manuscript), histone 2B (NP_003514.2; aa 1–126), Interferon-α (NP_076918.1|; aa 24–189), Interferon-λ (NP_742152.1|; aa 20–200), Interferon-ω (NP_002168.1| ; 24–195), Interferon-γ (NP_000610.2|; aa 24–166), GMCSF (NP_000749.2|; aa 15–144), GAD65 (NP_000809.1|; aa 1–585), aquaporin-4 (NP_001641.1|; aa 2–323), tyrosine hydroxylase (NP_000351.2|; aa 2–497) and glial fibrillary acidic protein (NP_002046.1|; aa 2–432). All antigens used in this study were cloned in-frame between *BamH1* and *Xho1* sites in the previously described pREN2 vector containing an N-terminal FLAG epitope tag [17]. The primer adaptor sequences used to amplify these genes are provided in Table S1. DNA midiprep DNA for each plasmid construct was then prepared (Qiagen) and the correct sequence in each plasmid was confirmed by DNA sequencing.

Six of the major SLE Ruc-antigen fusion proteins (La, Sm-D3, Ro52, Ro60, RNP-A, RNP-70k) produced from transfected mammalian cells were also analyzed by Western blot. Production of Ruc-antigens involved using COS1 cells that were maintained at 5% CO_2_, 37°C in high glucose DMEM (HyClone) supplemented with 10% fetal calf sera and 2 mM L-glutamine. For recombinant protein expression, COS1 cells were transfected with a mixture of 1–2 µg of each pREN2-antigen plasmid along with FuGENE 6 (Roche) reagent as previously described [18]. Forty-eight hours after transfection, tissue culture media was removed and the cell layer harvested in SDS PAGE sample buffer. The proteins were resolved by 4–12% Tris-Glycine PAGE electrophoresis and then electrotransfered to nitrocellulose. Following blocking with bovine serum albumin, a horseradish peroxidase-conjugated anti-FLAG monoclonal antibody (Sigma) was used to directly detect the FLAG-tagged Ruc-antigens by enhanced chemiluminescence (ECL) reagents (Figure S1). Western blotting revealed that all six Ruc-antigen fusion proteins migrated at their predicted molecular weight and appeared intact with little evidence of proteolytic processing.

For production of Ruc-antigen fusion proteins for LIPS, the same transfection protocol was followed. However for harvesting the Ruc-antigen fusion, the COS1 cells were first rinsed in PBS and then scrapped in 1.4 ml of cold lysis buffer composed of 50 mM Tris, pH 7.5, 100 mM NaCl, 5 mM MgCl_2_, 1% Triton X-100, 50% glycerol and protease inhibitors (Mini protease inhibitor cocktail, Roche). The cell lysate was sonicated, centrifuged and the cleared supernatants were collected and used immediately or stored at −80°C as described [18].

### LIPS analysis

LIPS was performed in a 96-well plate format as described [18]. For each test, 1 µL equivalent of serum was used. Additional sera dilutions were required for anti-Ro60 assays. Plates were washed on a Tecan Hydroflex (Tecan Systems, San Jose, CA), and light units (LU) were measured in a Berthold LB 960 Centro luminometer (Berthold Technologies, Germany) using coelenterazine mix (Promega, Madison, WI). Light unit data were the average of at least two independent experiments.

### Statistical analysis

GraphPad Prism software (San Diego, CA) was used for statistical analysis. Mann-Whitney *U*-tests were used to compare antibody titers among the different clusters. Cutoffs for sensitivity and specificity were determined using optimal separation based on receiver operator characteristics (ROC). Fisher's exact test was employed to evaluate differences in autoantibody frequency between clusters.

### Heatmap assembly and determination of autoantibody enriched clusters

To compare autoantibody titers between different antigens, a colored heatmap based on the *Z* score of each titer was employed. First, a cutoff was calculated based on the mean plus three standard deviations of the seronegative healthy controls for each antigen. This cutoff was subtracted from the autoantibody titer measured for each patient, and the resulting value was divided by the standard deviation of the control cluster to yield the *Z* score. Patients were then color coded by the number of standard deviations above the calculated cutoff.

## Results

### Detection of autoantibodies against the major SLE antigens by LIPS

Based on the fact that solution phase immunoassays provide more discriminatory quantitative antibody profiles than solid phase ELISA [14], [19], a panel of seven, known nuclear and extractable SLE antigens produced in mammalian cells was evaluated in a pilot cohort of 76 SLE patients and 18 healthy controls. These antigens included Sm-D3, RNP-A, RNP-70k, histone 2B, La, Ro52 and Ro60. For each antigen, the optimal separation between the SLE and control groups were determined using Receiver Operator Characteristics (ROC) and the sensitivity and specificity was calculated (Table 1). The dynamic range (95% CI) and the statistical difference (Mann Whitney *U* test) was determined for each antigen between the SLE and control samples (Table 1). From LIPS testing, antibodies against the RNP-70k antigen demonstrated the greatest sensitivity in the antigen panel with 71% sensitivity and 94% specificity (Table 1). The related RNP-A protein was only 59% sensitive (94% specific), and detected SLE patients who were all also seropositive for RNP-70k autoantibodies by LIPS. Detecting antibodies against the Sm antigen in SLE also showed a sensitivity of 59% and a specificity of 94%. Analysis of autoantibodies against Ro52 and Ro60, comprising the SSA antigen, using immunodominant protein fragments from each protein demonstrated 50% and 57% sensitivity, respectively and autoantibodies against SSB/La were detected in 49% of the SLE patients (Table 1). Finally, in the LIPS assay, anti-histone 2B autoantibodies were the least sensitive, detecting only 42% of the SLE patients, but did not add to the overall diagnostic performance because seropositive patients were already positive for at least one of the other nuclear antigens by LIPS. Overall, a six antigen panel consisting of Sm-D3, RNP-A, RNP-70k, La, Ro52 and Ro60 detected at least one statistically significant SLE antibody in 88% (67/76) of this SLE pilot cohort. As shown in Table 1, LIPS profiling of antibodies against these antigens demonstrated large dynamic ranges of detection, which were often 10–200 fold higher in SLE compared to the normal controls. These findings suggest that LIPS provides highly discriminatory detection of antibodies for SLE diagnosis and potentially even for disease stratification and symptoms research.

**Table 1:** LIPS diagnostic autoantibody characteristics in SLE.

	Sensitivity (%)	Specificity (%)	Mean titer HC (95% CI)	Mean titer SLE (95% CI)	*P*
**Core SLE antigens**					
Ro52	50	89	90,000 (−55,00–236,00)	408,000(251,000–565,000)	<0.001†
Ro60	57	83	178,000 (−169,000–525,000)	898,000(609,000-1.2×10^6^)	0.0019†
La	49	83	126,000 (−104,500–356,000)	265,000 (100,000–430,000)	0.0193†
Sm	59	94	3,000 (2,000–4,200)	26,000 (9,500–42,000)	<0.0001†
RNP-A	59	94	16,000 (2,100–29,000)	121,000 (39,000–203,000)	<0.0001†
RNP-70k	71	94	7,900 (5,000–11,000)	103,000 (69,000–137,000)	<0.0001†
Histone 2B	42	89	22,000 (17,000–27,000)	50,000 (39,000–61,000)	0.0047†
Overall sensitivity of core antigens*	**88**	**72**			
**Interferons**					
IFN-α	13	94	1,600 (1,300–1,900)	2,900 (1,500–4,200)	0.1118
IFN-ω	29	100	2,600 (2,400–2,700)	7,400 (285–15,000)	0.0030†
IFN-λ	17	100	19,000 (17,000–22,000)	66,000 (−14,000–146,000)	0.0345†
IFN-γ	10	94	7,200 (5,200–9,100)	7,500 (5,800–9,200)	0.5708
Overall prevalence of anti-IFN antibodies	**42**	**94**			
**Neuronal proteins**					
GAD65	30	89	2,500 (2,000–3,000	5,000 (3,500–6,500)	0.0739
AQP-4	12	94	4,900 (3,100–6,700)	9,400 (4,900–14,000)	0.1441
TH	30	89	5,000 (2,000–8,000)	10,000 (5,100–15,000)	0.1739
GFAP	16	100	13,000 (10,800–15,000)	22,000 (16,000–28,000)	0.0763
Overall prevalence of anti-neuronal antibodies	**47**	77			

*Excluding Histone 2B.

† Statistically significant, *P*<0.05.

### Anti-interferon and anti-neuronal autoantibodies in Lupus patients

Based on our previous ability to detect anti-cytokine and neuronal autoantibodies in other diseases by LIPS [16], [20], [21], a select panel of Ruc-antigen fusion proteins was evaluated in the pilot cohort. Screening for autoantibodies against 4 different interferon proteins including IFN-α, IFN-λ, IFN-ω, and IFN-γ revealed statistically significant autoantibodies to all four interferons in SLE patients compared to controls (Table 1). Anti-IFN-ω autoantibodies were the most common, and were detected in 29% (22/76) of patients (Table 1). In contrast, anti-IFN-λ, anti-IFN-α and anti-IFN-γ autoantibodies were seropositive in fewer patients with sensitivities of 17%, 13% and 10%, respectively. Additional screening for other cytokines revealed only a few low titer autoantibodies in SLE patients (e.g. anti-GMCSF autoantibodies in 4% of the SLE patients). Of note, several individual SLE patients showed quite high titer anti-interferon autoantibodies that were 100 times higher than the control group. Overall, 42% (32/76) of patients demonstrated reactivity toward at least one interferon. These results also suggest that anti-IFN-ω and other anti-interferon autoantibodies are quite common in SLE.

Autoantibody responses were also evaluated against a number of neurological antigens, including glutamic acid decarboxylase (GAD-65), aquaporin-4 (AQP-4), tyrosine hydroxylase (TH) and glial fibrillary acidic protein (GFAP). Seropositive autoantibody responses against GAD-65 and TH autoantigens were the most frequent, occurring in 30% of the SLE patients (Table 1). Anti-GFAP and anti-AQP4 autoantibodies were less common, and were detected in 16% and 12% of patients, respectively. The calculated specificity of these LIPS antigen tests ranged between 89% and 100%, demonstrating that these neuronal autoantibodies are rarely found in the healthy controls. Overall, almost half of the SLE patients (36/76, 47%) had autoantibodies against this panel of four neuronal antigens.

### Two major autoantibody clusters in SLE

**Figure 1:**
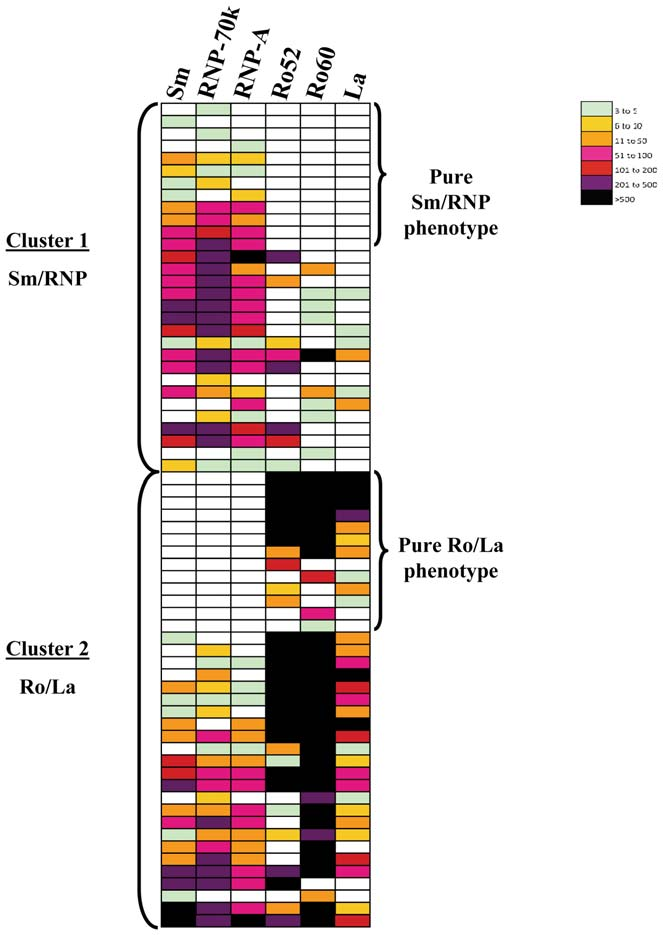
SLE autoantibody clusters in the pilot cohort. A colored heat map was used to visualize the autoantibody titers in the SLE patients. Autoantibody titers were transformed to *Z* scores as described in the Materials and Methods and color coded as indicated by the scale at right, in which signal intensities from green to black indicate high and low titers, respectively. To segregate the SLE patients into clusters, the relative ratio (RR) of the sum titer of Sm-D3, RNP-A and RNP-70k divided by the sum titer of Ro52, Ro60 and La autoantibodies was calculated for each patient. Patients with a RR≥1 were assigned to the Sm/RNP cluster (top panel) while patients with a RR<1 were assigned to the Ro/La cluster (bottom panel). Patients with a pure Sm/RNP or pure Ro/La phenotype are indicated.

To see if particular clusters or patterns of autoantibodies were present, a colored heat map based on the *Z* score of each antibody titer above the mean of the seronegative control samples was used to compare autoantibody titers between different antigens in the different patients in the pilot cohort (Fig. 1). Following the generation of this antibody titer-based heatmap, two major patterns emerged. One subset of patients showed immunoreactivity predominately to Sm-D3, RNP-A or RNP-70k, but less pronounced or no immunoreactivity at all against Ro52, Ro60 or La proteins. Conversely, a second subset showed a dominant pattern of reactivity against Ro52, Ro60 and/or La autoantigens, but had significantly less or no immunoreactivity against Sm-D3, RNP-A or RNP-70k. Interestingly, some patients within these groups were mutually exclusive of each other. For example, 14% (11/76) of the SLE patients showed pure Sm/RNP reactivity without immunoreactivity against Ro or La antigens, while 17% (13/76) of the SLE patients demonstrated a pure Ro/La reactivity without immunoreactivity against Sm, RNP-A or RNP-70k (Fig. 1). To further segregate the patients with a mixed Sm/RNP and Ro/La autoantibody phenotype, a straightforward algorithm was utilized. For determining cluster assignment, the relative ratio (RR) of autoantibody titers against the sum titer of Sm-D3, RNP-A and RNP-70K was compared to the sum titer for Ro52, Ro60 and La, whereby patients with RR≥1 were assigned to a Sm/RNP cluster, while patients with a RR<1 were assigned to a Ro/La cluster. Based on these criteria, 41% (31/76) of the SLE samples showed a Sm/RNP cluster phenotype, while 47% (36/76) showed a Ro/La phenotype (Fig. 1). Additionally, a small subset of SLE patients (12%, 9/76) displayed no significant seropositive autoantibody responses to any of these 6 antigens tested. Further analysis of these autoantibody groups using Fisher's exact test revealed no statistical difference in the frequency of IFN or neuronal antibodies between the Sm/RNP and Ro/La clusters (data not shown). Taken together, these results suggest that 88% of the SLE patients from the pilot cohort show either a prominent Sm/RNP or Ro/La autoantibody cluster phenotype.

### Two major autoantibody clusters and other autoantibodies in a second SLE cohort

To determine if the two major autoantibody clusters identified in the pilot cohort showed a relationship with documented clinical symptoms, a new independent cohort consisting of 15 controls and 129 SLE patients was obtained. The new cohort had female to male ratio of 13∶1, and the average age at diagnosis was 31±12.1 years. Similar to other published studies with SLE patients, the most frequent clinical manifestations in this SLE cohort were musculoskeletal symptoms (37%) and mucocutaneous symptoms (36%). Eleven patients (9%) were classified as serologically active but clinically quiescent, and eleven patients (9%) were classified as quiescent. Evaluation of six of the major SLE antigens (RNP-A, RNP-70k, Sm-D3, La, Ro52 and Ro60) with LIPS disclosed a wide dynamic range of detectable antibody titers with significant differences in the GMTs for each antigen between the SLE patients and the control group (Table 2). Anti-RNP-70k autoantibodies again showed the highest sensitivity and detected autoantibodies in 85% of SLE patients. Anti-Sm and anti-La autoantibodies were detected in 65% and 71% of the SLE patients, respectively (Table 2) and anti-Ro52 and anti-Ro60 autoantibodies were each found in 51% of the SLE patients (100% specificity).

**Table 2:** Sensitivity and specificity of Ruc-antigen fusions in an independent validation cohort.

	Sensitivity (%)	Specificity (%)	Mean titer HC (95% CI)	Mean titer SLE (95% CI)	*P*
**Core SLE antigens**					
Ro52	51	100	10,000 (6,000–14,000)	895,000 (636,000-1.1×10^6^)	<0.0001^†^
Ro60	51	100	5,000 (3,400–7,200)	1.2×10^6^ (915,000-1.5×10^6^)	0.0004^†^
La	71	93	4,700 (3,300–6,100)	464,000 (294,000–634,000)	<0.0001^†^
Sm	65	100	4,800 (4,200–5,400)	77,000 (49,000–105,000)	<0.0001^†^
RNP-A	55	100	12,600 (8,200–17,000)	276,000 (212,000–339,000)	0.0017^†^
RNP-70k	85	100	28,800 (24,800–32,800)	559,000 (440,000–678,000)	<0.0001^†^
Overall sensitivity of core antigens	**98%**	**93%**			
**Cytokines**					
IFN-α	12	100	2,000 (1,600–2,500)	88,000 (66–175,000)	0.0686
IFN-ω	38	100	1,900 (1,700–2,000)	7,800 (2,500–13,000)	0.0003^†^
**Neuronal proteins**					
AQP-4	5	93	10,600 (2,000–19,000)	27,000 (−9,100–62,000)	0.7162
GAD65	5	93	4,300 (2,700–6,000)	4,200 (3,200–5,300)	0.7951
GFAP	17	100	7,700 (5,100–10,000)	21,000 (2,200–40,000)	0.1930
TH	8	93	11,600 (3,700–20,000)	15,000 (10,200–20,000)	0.2841

**Figure 2:**
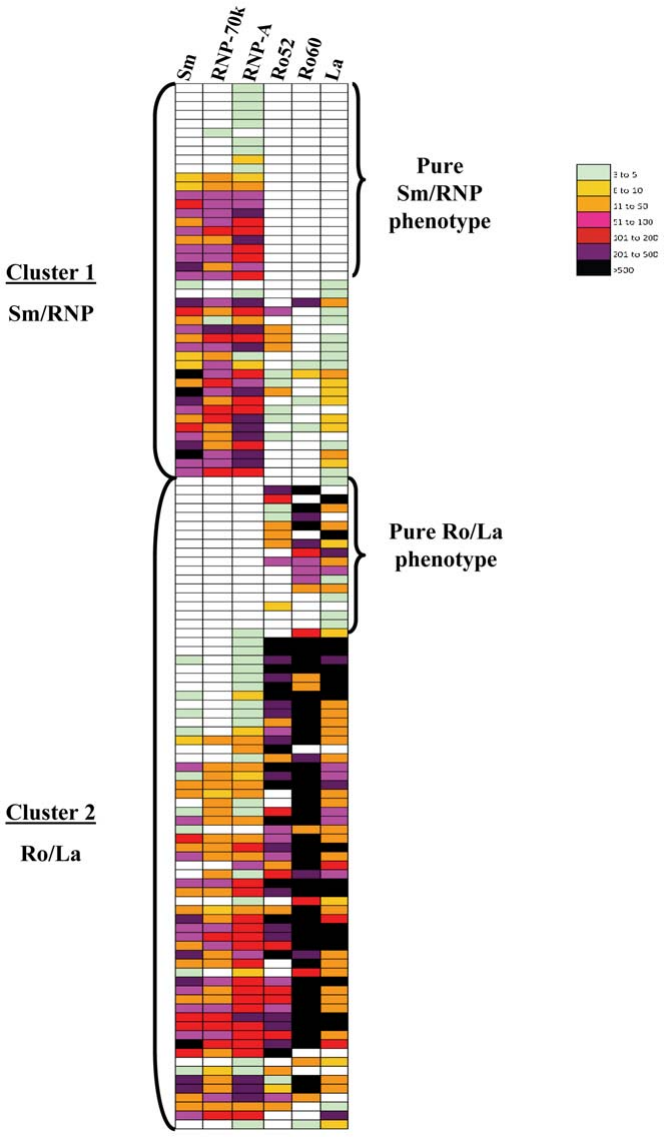
Validation of two major autoantibody clusters in SLE. A second SLE cohort was analyzed for autoantibody titers. A heatmap was again constructed and antibody clusters were determined as in Figure 1. Similar to the prevalence seen in the first cohort, 47% of the SLE samples showed a Sm/RNP cluster phenotype and 51% showed a Ro/La cluster phenotype.

Similar to the pilot cohort, a colored heat map based on the Z score of each antibody titer data in the second SLE cohort also showed the two major autoantibody clusters. Using the segregation algorithm developed for the pilot cohort, we observed in the validation cohort that 47% (61/129) of the SLE samples showed a Sm/RNP cluster phenotype, 51% (66/129) showed a Ro/La cluster phenotype and 2% (2/129) displayed no significant autoantibody responses to any of the antigens tested (Fig. 2). Using the Fisher's exact statistical test, the frequency of six disease manifestations, including nephritis, serositis and musculoskeletal, mucocutaneous, hematologic and CNS symptoms, were compared between the two clusters (Table 3). Surprisingly, the only significant difference between the two clusters was the frequency of serositis, inflammation occurring in serous membranes surrounding body organs, which correlated with the Sm/RNP cluster (*P* = 0.0022). Furthermore, analysis of the two clusters revealed that there were no differences in the proportion of SACQ or quiescent patients and no difference in the average SLEDAI score.

**Table 3:** Percentage of patients in each cluster with specific clinical manifestations and autoantibodies.

	Ro/La	Sm/RNP	*P*
**Clinical Symptoms**			
CNS	7	2	0.20
Musculoskeletal	30	44	0.09
Mucocutaneous	39	33	0.58
Nephritis	37	25	0.18
Serositis	0	13	**0.002**
Hematological	18	13	0.47
SACQ	7	10	0.75
Quiescent	4	13	0.11
**Autoantibodies**			
IFN-α	6	17	0.06
IFN-ω	41	35	0.59
TH	8	10	0.76
AQP-4	5	5	1.0
GAD65	2	6	0.20
GFAP	15	17	0.81

The frequency of IFN and neuronal protein targets were also evaluated by autoantibody cluster. Within the entire cohort, anti-IFN-ω autoantibodies were the most prevalent (38%) and anti-IFN-α autoantibodies were observed in only 12% of patients. With the exception of GFAP, the other neurological antigens were less commonly detected in this second group compared to the pilot cohort (Table 2). Anti-TH autoantibodies were observed in 8% of patients, while anti-AQP4 and anti-GAD65 autoantibodies were each only observed in 5% of patients. By cluster, however, there were no significant differences in the frequency of anti-neuronal and anti-IFN autoantibodies between patients with Sm/RNP or Ro/La cluster phenotype (Table 3).

### Correlation of clinical characteristics with autoantibody titers

In addition to analyzing clinical correlations between the two identified autoantibody clusters, individual autoantigens were also inspected to evaluate whether they correlated with clinical symptoms. While many autoantibodies did not correlate with particular clinical symptoms (Table 4), anti-RNP-70k autoantibodies were found to be more prevalent in patients with musculoskeletal manifestations than those without symptoms (Fisher's exact test 94% v. 79%; *P* = 0.059). Patients with mucocutaneous manifestations were also more frequently observed with anti-Sm (81% v. 56%; *P* = 0.007), anti-RNP-A (68% v. 46%; *P* = 0.01) and anti-RNP-70k (96% v. 78%; *P* = 0.01) autoantibodies in comparison to those without these symptoms. Interestingly, analysis of the anti-IFN-ω within the cohort at large revealed that IFN-ω seropositive SLE patients were more likely to have positive anti-Sm (Fisher's exact test, 80% v. 56%; *P* = 0.0079) and anti-IFN-α (22% v. 5%; *P* = 0.0041) autoantibodies compared to IFN-ω seronegative SLE patients. Moreover, the GMT of anti-Sm autoantibodies was also significantly higher in the anti-IFN-ω positive group (Student's *t*-test, *P* = 0.0037). Anti-IFN-α autoantibodies were observed in only 12% of patients and were more common in female v males (Fisher's exact test, 44% v. 9%; *P* = 0.0108).

**Table 4:** Percentage of autoantibody positive patients by a given clinical manifestation.

	CNS			Musculoskeletal			Mucocutaneous			Nephritis		
	−	+	P	−	+	P	−	+	P	−	+	P
Sm	64	83	0.66	59	75	0.08	56	81	**0.007**	64	68	0.69
RNP-A	53	83	0.21	51	60	0.36	46	68	**0.01**	55	54	1.0
RNP-70k	85	83	1.0	79	94	**0.02**	78	96	**0.01**	81	93	0.11
Ro52	50	67	0.68	52	50	0.85	48	57	0.36	47	59	0.26
Ro60	50	83	0.21	57	42	0.10	50	53	0.85	45	63	0.06
La	71	83	0.67	73	69	0.68	70	74	0.69	69	76	0.53
IFN-α	12	0	1.0	1	14	0.57	12	11	1.0	15	5	0.14
IFN- ω	37	50	0.67	38	37	1.0	37	38	1.0	34	46	0.24
TH	9	0	1.0	11	4	0.32	11	4	0.33	6	15	0.10
AQP-4	5	0	1.0	5	4	1.0	5	4	1.0	5	5	1.0
GAD65	3	16	0.22	2	6	0.36	5	2	1.0	5	2	1.0
GFAP	15	50	0.06	20	10	0.22	20	11	0.22	14	22	0.30

	Hematological			SACQ			Quiescent		
	−	+	P	−	+	P	−	+	P
Sm	65	65	1.0	67	45	0.19	67	36	**0.04**
RNP-A	53	60	0.63	56	36	0.34	58	18	**0.02**
RNP-70k	83	90	0.73	86	64	0.06	88	45	**0.002**
Ro52	48	65	0.22	53	27	0.12	54	18	**0.02**
Ro60	48	65	0.22	52	45	0.76	53	27	0.12
La	70	75	0.79	73	86	0.29	73	55	0.29
IFN-α	12	10	1.0	9	36	**0.02**	12	9	1.0
IFN- ω	38	35	0.80	36	45	0.53	38	27	0.53
TH	9	5	1.0	8	9	1.0	9	0	0.59
AQP-4	5	5	1.0	5	0	1.0	5	0	1.0
GAD65	5	0	1.0	4	0	1.0	4	0	1.0
GFAP	17	10	0.53	16	18	1.0	15	27	0.38

Eleven of 129 patients in the second cohort were classified as SACQ, a subgroup of SLE defined as having clinically quiescent symptoms despite increased anti-dsDNA and/or low serum complement levels using conventional clinical assays. While there was no difference in the frequency of seropositivity for anti-IFN-ω autoantibodies between the patients with and without SACQ classification, the SACQ patients had a significantly higher frequency of anti-IFN-α autoantibodies, with 4/11 seropositive (36% v. 9%; *P* = 0.024) (Table 4). Together these results suggest that the SACQ patient subgroup has a unique autoantibody profile that may play a role in their relatively less severe clinical symptoms. Interestingly, there was a lower frequency in Ro52, Sm-D3, RNP-A and RNP-70k seropositivity in the quiescent group compared to the rest of the cohort (Table 4).

Autoantibody profiles were also evaluated among the different SLE ethnic and age groups. From this analysis, African-American SLE patients were more frequently positive for anti-Sm (76% v. 54%; *P* = 0.0187) and anti-RNP-A (75% v. 37%; *P* = 0.0001) autoantibodies compared to Caucasians (Table 5). Overall, the African Americans showed larger number of samples belonging to the cluster 1 enriched phenotype compared to the Caucasians, but the difference was not statistically significant (data not shown). Anti-IFN-ω autoantibodies were also most prevalent in African Americans SLE patients compared to both Caucasians (54% v. 29%; *P* = 0.0131) and Asians (54% v. 0; *P* = 0.024). Autoantibody profiles were also examined by age group. Middle age range patients (20–40 years of age) were more frequently positive for anti-RNP-A autoantibodies compared to those >40 years of age (60% v. 34%; *P* = 0.0280), and more frequently positive for anti-Ro52 autoantibodies than individuals less than 20 years of age (61% v. 36%; *P* = 0.0368) and greater than 40 years of age (61% v. 37%; *P* = 0.0472) (Table 6).

**Table 5:** Percentage of seropositive patients by ethnicity.

	C	AA	A	P
Sm	54	76	83	0.01*
RNP-A	37	75	66	0.0001*
RNP-70k	78	90	100	
Ro52	53	54	50	
Ro60	45	54	67	
La	63	79	83	
IFN-α	14	12	0	
IFN-ω	29	54	0	0.01*, 0.02**
TH	6	8	17	
AQP-4	5	6	0	
GAD65	3	4	17	
GFAP	11	23	0	

**C**: Caucasian; **AA**: African American; **A**: Asian. Significant differences in the frequency of autoantibody titers:

*White v. African American;

**African American v Asian.

**Table 6:** Percentage of seropositive patients by age group.

	<20	20–40	>40	P
Sm	72	69	48	
RNP-A	60	60	34	0.028**
RNP-70k	88	85	79	
Ro52	36	61	37	0.037*, 0.047**
Ro60	44	57	41	
La	80	75	55	
IFN-α	16	9	14	
IFN-ω	52	36	31	
TH	24	4	7	0.007*
AQP-4	4	5	3	
GAD65	8	1	7	
GFAP	12	20	10	

Significant differences in the frequency of autoantibody titers:

*<20 v. 20–40;

**20–40 v. >40.

Lastly, we also examined whether the frequency of the major SLE autoantibodies correlated with dsDNA autoantibody status. Using the standard clinical assay, 58% (75/129) of SLE patients in the cohort were positive for anti-dsDNA autoantibodies. Anti-dsDNA positive patients were more frequently positive than seronegative patients for anti-Sm (73% v. 53%; *P* = 0.022), anti-Ro60 (61% v. 35%; *P* = 0.006) and anti-La (79% v. 61%; *P* = 0.0428) autoantibodies (Table 7). Additionally, anti-IFN-ω (48% v. 24%; *P* = 0.0085) and anti-TH (12% v. 2%; *P* = 0.048) autoantibodies were all more prevalent in patients with dsDNA autoantibodies than those without. Taken together, these results suggest that SLE patients seropositive for Sm, Ro60, La, INF-ω and anti-TH autoantibodies are also often seropositive for anti-dsDNA.

**Table 7:** Percentage of seropositive patients by anti-dsDNA antibody status.

	−	+	P
Sm	53	73	**0.022**
RNP-A	45	61	0.100
RNP-70k	78	89	0.127
Ro52	43	59	0.103
Ro60	35	61	**0.006**
La	61	79	**0.043**
IFN-α	10	12	0.779
IFN-ω	24	48	**0.008**
TH	2	12	**0.048**
AQP-4	8	3	0.221
GAD65	4	4	1.0
GFAP	16	16	1.0

### A simple LIPS mixture test for SLE diagnosis

**Figure 3:**
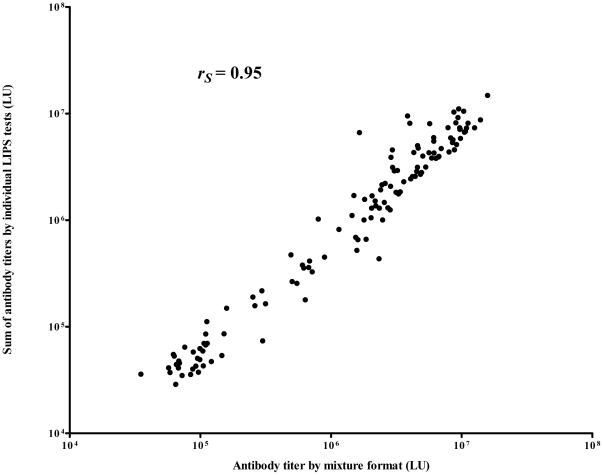
Comparison of LIPS mixture test with the sum of the individual tests for SLE diagnosis. Antibody titer data from the LIPS mixture test was plotted against the sum of the titer of the individual Ro52, Ro60, La, Sm-D3, RNP-A and RNP-70k autoantibody titers. Each circle represents an individual patient. Using the Spearman rank test, the correlation between the two tests was *R* = 0.95.

One major advantage of LIPS is the ability to test mixtures of antigens, which simplifies data collection and often improves the overall performance of the assay [22], [23]. Based on the results from the individual LIPS tests for the six different nuclear antigens, a mixture format using the 6 autoantigen panel of Sm-D3, RNP-A and RNP-70k, Ro52, Ro60, and La was evaluated with 1 µL of serum from the different clinical samples. The results of testing using the mixture demonstrated 83% sensitivity and 93% specificity for cohort 2 (Fig. 3). As found with testing the autoantigens individually, one healthy control was seropositive in the mixture format and this was most likely due to high anti-La antibody titers (data not shown). Comparison of the mixture results with the sum of the individual antibody titers determined previously revealed that the titer values strongly correlated (Spearman Rank *R* = 0.95 (Fig. 3). These results suggest that the LIPS mixture format is comparable to the individual antibody tests and thus provides a simplified format for the detection of SLE autoantibodies.

## Discussion

Autoantibodies are important elements in both the diagnosis and monitoring of SLE, as some antibodies appear before the onset of clinical symptoms and others are associated with specific clinical manifestations [1], [24]. In this study, we employed the liquid phase LIPS assay, based on luciferase-tagged antigens, to generate quantitative autoantibody profiles against major SLE autoantigens, IFNs and neuronal proteins. Remarkably, we found two major autoantibody clusters in SLE consisting of a Sm/RNP cluster and a Ro/La cluster. Using our highly sensitive LIPS assay and a simple segregation algorithm based on relative autoantibody titers, 88% of the pilot cohort and 98% of a second cohort showed roughly similar numbers of SLE patients belonging to either one of these two autoantibody clusters. Another intriguing feature is that some patients had immunoreactivity to the Sm/RNP cluster without immunoreactivity against antigens in the Ro/La cluster, while other SLE patients demonstrated immunoreactivity to the Ro/La cluster without immunoreactivity against Sm/RNP antigens. Evaluation of the genetic backgrounds of these select groups of patients with “pure” autoantibody phenotypes might prove to be highly informative.

A previous study by To and Petri identified three autoantibody clusters from analysis of seven antigens, including Sm, RNP, dsDNA, lupus anticoagulant (LAC), cardiolipin (CL), Ro and La [11]. In their study, the researchers used standard immunoassays for evaluating autoantibodies and a *K*-means clustering bioinformatics strategy for analysis. [11]. Three autoantibody clusters were identified and assignment to each cluster was based on *K*-means clustering analysis. This resulted in grouping of patients with similar autoantibody profiles. Their clusters represented enrichment of particular autoantibodies. For example, in their Sm/RNP cluster, only a subset of patients, 22.2% and 39.5%, showed immunoreactivity against Sm and RNP, respectively. In contrast, our clustering analysis was based on the quantitative measurement of autoantibody titers using defined recombinant proteins. Despite these differing approaches, our results confirm two of the three clusters first observed by To and Petri.

After analyzing six antigens with LIPS in our study, ∼90% of the SLE patients were found to belong to either the Sm/RNP or Ro/La cluster. Importantly, every SLE patient within a particular cluster always had immunoreactivity against the relevant antigens. The clinical significance of our clustering approach revealed that the Sm/RNP cluster was often associated with the presence of serositis. While an association between anti-Sm autoantibodies and serositis has previously been observed in pediatric lupus populations [25], the association in adult SLE patients has not been reported. More extensive clinical data, such as the prevalence of additional hematological abnormalities and sicca symptoms were not collected here yet may yield further insight into the clinical significance of the observed autoantibody clusters. The Ro/La cluster observed here in SLE patients is similar to that of Sjögren's Syndrome, in which 75% of the patients also show autoantibodies against Ro52, Ro60 and La [26]. Since 5/6 autoantigens used for clustering represent known RNA binding proteins, further understanding the details of these RNA-binding proteins and pathways may lead to additional insights into the mechanisms and/or triggers involved in both SLE and SjS.

Based on the available patient information, two interesting clinical correlations emerged from our analysis of LIPS serological profiles outside the autoantibody clusters. First, within the cohort at large, anti-RNP-70k autoantibodies were significantly associated with musculoskeletal manifestations, and anti-RNP-70k, RNP-A and Sm autoantibodies with mucocutaneous symptoms. To our knowledge, the association of these autoantibodies with mucocutaneous symptoms has not yet been reported, but anti-RNP autoantibodies have previously been associated with musculoskeletal symptoms in African-American patients [27] and with Raynaud's phenomenon in Chinese patients [28]. While an association between the Ro/La patient cluster and an increased prevalence of sicca symptoms was observed by To and Petri [11], the prevalence of sicca symptoms was not available for this cohort. Since primary SjS patients also have high titer autoantibodies to Ro52, Ro60 and La, future large scale genetic studies may provide additional clues to the similarities and differences between SjS and SLE patients within the Ro/La autoantibody cluster. The lack of clinical correlates with our autoantibody SLE data also suggests that these major diagnostic SLE antigens are likely not to be informative for identifying patients with pulmonary, renal, CNS, and hematologic symptoms. It is likely that the inclusion of other organ-specific antigens would be needed to identify clinically relevant subtypes. For example, α-actinin, might be incorporated in LIPS testing to identify patients with lupus nephritis [29]. Future studies expanding the antigen panels in the LIPS format to more comprehensively target specific organ systems may yield additional insight into the full range of lupus symptomatology.

Elevated levels of interferon proteins and/or activity are associated with up-regulation of interferon-inducible genes and are thought to contribute to the pathophysiology of SLE [30]. Seropositive anti-IFN-ω autoantibodies were detected in 29% and 38% of the SLE patients in the pilot and second cohort, respectively. These autoantibodies have been observed in previous studies of SLE but at much lower frequencies, most likely due to limitations of the detection methods employed [31], [32], [33], [34]. Anti-IFN-ω autoantibodies have been reported in several other autoimmune diseases, including in late-onset myasthenia gravis, and are associated with mutations in the autoimmune regulator (*AIRE*) gene in autoimmune polyendocrine syndrome type 1 [35]. Together, these observations suggest that anti-IFN-ω autoantibodies may play a role in immune mediated disease. Anti-IFN-ω positive patients also had significantly higher titers of anti-Sm, RNP-A and RNP-70k autoantibodies and were more frequently positive for anti-dsDNA in comparison to those patients without anti-IFN-ω autoantibodies, suggesting an overall increased autoantibody burden in this group.

In our study, a significantly higher prevalence of anti-IFN-α autoantibodies was detected in the SACQ group in comparison to the rest of the SLE cohort. These results are consistent with previous studies demonstrating an inverse relationship between disease activity and the presence of anti-IFN-α autoantibodies [31], [34]. However, unlike previous groups who did not detect antibody titer differences in core SLE antigens between anti-IFN-α positive and negative groups [31], we detected significantly higher anti-Sm and RNP-A titers in the anti-IFN-α positive group. These results suggest that despite their clinical phenotype, this subset of patients has a significantly higher autoantibody burden and supports recent data of a protective effect of anti-IFN-α antibodies in SLE [31]. Interestingly, a recent study demonstrated clinical efficacy for treating SLE with a monoclonal anti-IFN-α antibody therapy [36], [37]. These results are consistent with our findings that endogenous anti-IFN-α autoantibodies may dampen immune activation and be correlated with a favorable phenotype. Future studies of larger patient cohorts employing this simple LIPS test to detect anti-INF-α autoantibodies in longitudinal samples in parallel with clinical outcomes are needed to substantiate whether these autoantibodies have any role in modulating the clinical outcome of these patients.

The diagnosis of SLE by serology requires testing a battery of individual nuclear and extractable antigen tests which can be costly in terms of both time and money. One potential technical advance was our ability to test a mixture of SLE antigens quickly and cheaply with LIPS. In our study, a single LIPS test simultaneously evaluating six autoantigens demonstrated 83% sensitivity and 93% specificity. Ironically, our mixture employed only one autoantigen, Sm, which is part of the diagnostic criteria for SLE. It is highly likely that the inclusion of additional autoantigens could further improve this LIPS mixture format for the diagnosis of SLE and is consistent with our previous studies which utilized antigen mixtures to diagnose various infectious diseases [22], [23]. While combining SLE antigens in the LIPS mixture format is a practical approach for testing, it alone would not be sufficient to diagnose SLE because these autoantibodies are also present in other rheumatological diseases. Furthermore, additional validation and standardization is needed for the LIPS assay before it could be used clinically for diagnosis of SLE.

In summary, we report the presence of two distinct autoantibody clusters in SLE. The ability to segregate most SLE patients into two clusters was based on quantitative serological profiles and relatively simple analysis. One important clinical feature of the Sm/RNP cluster was an increased prevalence of serositis. In addition, we identified a link between anti-interferon-α autoantibodies and SACQ, a less severe form of SLE. Future studies with a larger number of SLE patients and with extensive clinical background, are needed to further validate the clinical significance of these findings.

## Supporting Information

Figure S1 **Western blotting of the six major SLE Ruc-antigen fusion proteins produced from transfected COS1 cells.**
(TIF)

Table S1 **Forward and reverse primers used for cloning of antigens used in this study.**
(XLS)

## References

[1] Rahman A, Isenberg DA (2008). Systemic lupus erythematosus.. N Engl J Med.

[2] Weckerle CE, Franek BS, Kelly JA, Kumabe M, Mikolaitis RA (2011). Network analysis of associations between serum interferon-alpha activity, autoantibodies, and clinical features in systemic lupus erythematosus.. Arthritis Rheum.

[3] Lockshin MD (2006). Sex differences in autoimmune disease.. Lupus.

[4] Powell JJ, Van de Water J, Gershwin ME (1999). Evidence for the role of environmental agents in the initiation or progression of autoimmune conditions.. Environ Health Perspect.

[5] Sestak AL, Furnrohr BG, Harley JB, Merrill JT, Namjou B (2011). The genetics of systemic lupus erythematosus and implications for targeted therapy.. Ann Rheum Dis.

[6] Chung SA, Taylor KE, Graham RR, Nititham J, Lee AT (2011). Differential genetic associations for systemic lupus erythematosus based on anti-dsDNA autoantibody production.. PLoS Genet.

[7] Harley JB, Alarcon-Riquelme ME, Criswell LA, Jacob CO, Kimberly RP (2008). Genome-wide association scan in women with systemic lupus erythematosus identifies susceptibility variants in ITGAM, PXK, KIAA1542 and other loci.. Nat Genet.

[8] Sherer Y, Gorstein A, Fritzler MJ, Shoenfeld Y (2004). Autoantibody explosion in systemic lupus erythematosus: more than 100 different antibodies found in SLE patients.. Semin Arthritis Rheum.

[9] Tan EM, Cohen AS, Fries JF, Masi AT, McShane DJ (1982). The 1982 revised criteria for the classification of systemic lupus erythematosus.. Arthritis Rheum.

[10] Hoffman IE, Peene I, Meheus L, Huizinga TW, Cebecauer L (2004). Specific antinuclear antibodies are associated with clinical features in systemic lupus erythematosus.. Ann Rheum Dis.

[11] To CH, Petri M (2005). Is antibody clustering predictive of clinical subsets and damage in systemic lupus erythematosus?. Arthritis Rheum.

[12] Malik S, Bruner GR, Williams-Weese C, Feo L, Scofield RH (2007). Presence of anti-La autoantibody is associated with a lower risk of nephritis and seizures in lupus patients.. Lupus.

[13] Somers E, Magder LS, Petri M (2002). Antiphospholipid antibodies and incidence of venous thrombosis in a cohort of patients with systemic lupus erythematosus.. J Rheumatol.

[14] Burbelo PD, Ching KH, Bren KE, Iadarola MJ (2011). Searching for biomarkers: humoral response profiling with luciferase immunoprecipitation systems.. Expert Rev Proteomics.

[15] Burbelo PD, Ching KH, Han BL, Bush ER, Reeves WH (2010). Extraordinary antigenicity of the human Ro52 autoantigen.. Am J Transl Res.

[16] Burbelo PD, Leahy HP, Issa AT, Groot S, Baraniuk JN (2009). Sensitive and robust luminescent profiling of anti-La and other autoantibodies in Sjogren's syndrome.. Autoimmunity.

[17] Burbelo PD, Goldman R, Mattson TL (2005). A simplified immunoprecipitation method for quantitatively measuring antibody responses in clinical sera samples by using mammalian-produced Renilla luciferase-antigen fusion proteins.. BMC Biotechnol.

[18] Burbelo PD, Ching KH, Klimavicz CM, Iadarola MJ (2009). Antibody profiling by Luciferase Immunoprecipitation Systems (LIPS).. J Vis Exp.

[19] Liu E, Eisenbarth GS (2007). Accepting clocks that tell time poorly: fluid-phase versus standard ELISA autoantibody assays.. Clin Immunol.

[20] Burbelo PD, Browne SK, Sampaio EP, Giaccone G, Zaman R (2010). Anti-cytokine autoantibodies are associated with opportunistic infection in patients with thymic neoplasia.. Blood.

[21] Burbelo PD, Seam N, Groot S, Ching KH, Han BL (2010). Rapid induction of autoantibodies during ARDS and septic shock.. J Transl Med.

[22] Burbelo PD, Leahy HP, Groot S, Bishop LR, Miley W (2009). Four-antigen mixture containing v-cyclin for serological screening of human herpesvirus 8 infection.. Clin Vaccine Immunol.

[23] Burbelo PD, Leahy HP, Iadarola MJ, Nutman TB (2009). A four-antigen mixture for rapid assessment of Onchocerca volvulus infection.. PLoS Negl Trop Dis.

[24] Arbuckle MR, McClain MT, Rubertone MV, Scofield RH, Dennis GJ (2003). Development of autoantibodies before the clinical onset of systemic lupus erythematosus.. N Engl J Med.

[25] Jurencak R, Fritzler M, Tyrrell P, Hiraki L, Benseler S (2009). Autoantibodies in pediatric systemic lupus erythematosus: ethnic grouping, cluster analysis, and clinical correlations.. J Rheumatol.

[26] Fox RI (2005). Sjogren's syndrome.. Lancet.

[27] Isenberg DA, Garton M, Reichlin MW, Reichlin M (1997). Long-term follow-up of autoantibody profiles in black female lupus patients and clinical comparison with Caucasian and Asian patients.. Br J Rheumatol.

[28] Tang X, Huang Y, Deng W, Tang L, Weng W (2010). Clinical and serologic correlations and autoantibody clusters in systemic lupus erythematosus: a retrospective review of 917 patients in South China.. Medicine (Baltimore).

[29] Deocharan B, Qing X, Lichauco J, Putterman C (2002). Alpha-actinin is a cross-reactive renal target for pathogenic anti-DNA antibodies.. J Immunol.

[30] Baechler EC, Batliwalla FM, Karypis G, Gaffney PM, Ortmann WA (2003). Interferon-inducible gene expression signature in peripheral blood cells of patients with severe lupus.. Proc Natl Acad Sci U S A.

[31] Morimoto AM, Flesher DT, Yang J, Wolslegel K, Wang X (2011). Association of endogenous anti-interferon-alpha autoantibodies with decreased interferon-pathway and disease activity in patients with systemic lupus erythematosus.. Arthritis Rheum.

[32] Nikpour M, Dempsey AA, Urowitz MB, Gladman DD, Barnes DA (2008). Association of a gene expression profile from whole blood with disease activity in systemic lupus erythaematosus.. Ann Rheum Dis.

[33] Slavikova M, Schmeisser H, Kontsekova E, Mateicka F, Borecky L (2003). Incidence of autoantibodies against type I and type II interferons in a cohort of systemic lupus erythematosus patients in Slovakia.. J Interferon Cytokine Res.

[34] von Wussow P, Jakschies D, Hartung K, Deicher H (1988). Presence of interferon and anti-interferon in patients with systemic lupus erythematosus.. Rheumatol Int.

[35] Meager A, Visvalingam K, Peterson P, Moll K, Murumagi A (2006). Anti-interferon autoantibodies in autoimmune polyendocrinopathy syndrome type 1.. PLoS Med.

[36] Merrill JT, Wallace DJ, Petri M, Kirou KA, Yao Y (2011). Safety profile and clinical activity of sifalimumab, a fully human anti-interferon alpha monoclonal antibody, in systemic lupus erythematosus: a phase I, multicentre, double-blind randomised study.. Ann Rheum Dis.

[37] Yao Y, Richman L, Higgs BW, Morehouse CA, de los Reyes M (2009). Neutralization of interferon-alpha/beta-inducible genes and downstream effect in a phase I trial of an anti-interferon-alpha monoclonal antibody in systemic lupus erythematosus.. Arthritis Rheum.

