# Supplementary figures and images for: Two Major Autoantibody Clusters in Systemic Lupus Erythematosus
Source: PLoS One. 2012 Feb 21;7(2):e32001. doi: 10.1371/journal.pone.0032001 (PMC3283706; doi:10.1371/journal.pone.0032001)

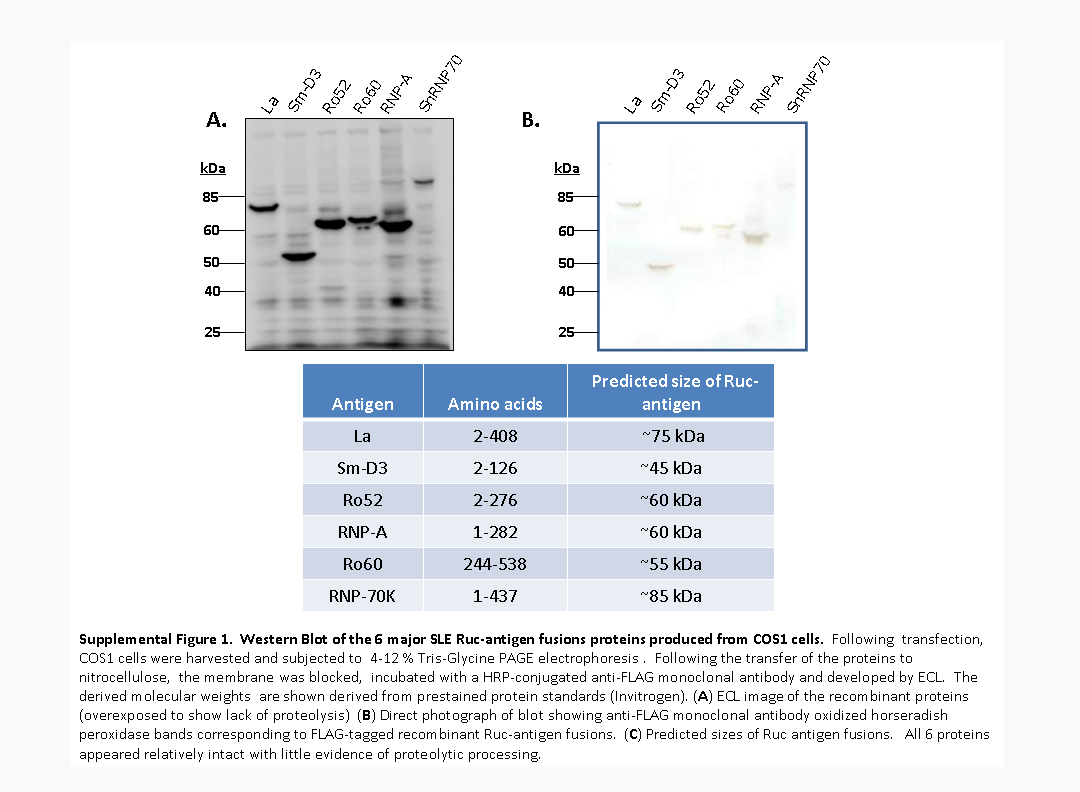

Supplement: Figure S1 — Western blotting of the six major SLE Ruc-antigen fusion proteins produced from transfected COS1 cells. (TIF) [file pone.0032001.s001.tif]
